# Integrating genomic resources to present full gene and putative promoter capture probe sets for bread wheat

**DOI:** 10.1093/gigascience/giz018

**Published:** 2019-01-31

**Authors:** Laura-Jayne Gardiner, Thomas Brabbs, Alina Akhunov, Katherine Jordan, Hikmet Budak, Todd Richmond, Sukhwinder Singh, Leah Catchpole, Eduard Akhunov, Anthony Hall

**Affiliations:** 1 Earlham Institute, Norwich Research Park, Norwich, NR4 7UZ, UK; 2 IBM Research, The Hartree Centre STFC Laboratory, Sci-Tech Daresbury, Warrington, WA4 4AD, UK; 3 Department of Plant Pathology, Kansas State University, Manhattan, KS, 66506, USA; 4 Department of Plant Sciences and Plant Pathology, Montana State University, Bozeman, MT, 59717, USA; 5 Roche Sequencing Solutions, 500 S Rosa Road, Madison, WI, 53719, USA; 6 CIMMYT, Calle Dr Norman E Borlaug, Ciudad Obregon, 85208, Mexico; 7 School of Biological Sciences, University of East Anglia, Norwich Research Park, Norwich, NR4 7TU, UK

**Keywords:** wheat, plant genomes, gene capture, promoter capture

## Abstract

**Background:**

Whole-genome shotgun resequencing of wheat is expensive because of its large, repetitive genome. Moreover, sequence data can fail to map uniquely to the reference genome, making it difficult to unambiguously assign variation. Resequencing using target capture enables sequencing of large numbers of individuals at high coverage to reliably identify variants associated with important agronomic traits. Previous studies have implemented complementary DNA/exon or gene-based probe sets in which the promoter and intron sequence is largely missing alongside newly characterized genes from the recent improved reference sequences.

**Results:**

We present and validate 2 gold standard capture probe sets for hexaploid bread wheat, a gene and a putative promoter capture, which are designed using recently developed genome sequence and annotation resources. The captures can be combined or used independently. We demonstrate that the capture probe sets effectively enrich the high-confidence genes and putative promoter regions that were identified in the genome alongside a large proportion of the low-confidence genes and associated promoters. Finally, we demonstrate successful sample multiplexing that allows generation of adequate sequence coverage for single-nucleotide polymorphism calling while significantly reducing cost per sample for gene and putative promoter capture.

**Conclusions:**

We show that a capture design employing an “island strategy” can enable analysis of the large gene/putative promoter space of wheat with only 2 × 160 Mbp probe sets. Furthermore, these assays extend the regions of the wheat genome that are amenable to analyses beyond its exome, providing tools for detailed characterization of these regulatory regions in large populations.

## Introduction

It is expensive to perform whole-genome sequencing to depths sufficient for confident variant calling, particularly in species with large genome sizes. To reduce this complexity and to make resequencing more cost effective, we can utilize approaches such as restriction site associated DNA sequencing [1], transcriptome sequencing [2], and sequence capture. Sequence capture typically combines probe hybridization to capture specific genome sequences in solution with sequencing of the captured fragments. The ability to design and implement specifically targeted probe sets has clear advantages for the analysis of variation across the genome. Sequence capture is used in human medicine for diagnosis and to inform treatment [3]; in crops such as rice, barley, soybean, and wheat to discover variants to aid agricultural improvement [4–6]; and in animals such as the pig *Sus scrofa* to identify genetic markers relating to animal health [7].

It is particularly expensive to perform whole-genome sequencing in wheat because of its vast genome, polyploid nature, and high repetitive content. The allohexaploid (AABBDD) wheat genome is 17 Gb in size and derived from 3 diploid progenitor genomes. The AA genome is from *Triticum urartu*, the BB is likely to be of the Sitopsis section (includes *Aegilops speltoides*), and the DD from *Aegilops tauschii* [8]. AABB tetraploids appeared <0.5 million years ago after an initial hybridization event [9]. It is thought that emmer tetraploid wheat developed from the domestication of such natural tetraploid populations. The hexaploid wheat that we have today formed ∼8,000 years ago by the hybridization of the unrelated diploid wild grass *A. tauschii* (DD genome) with the tetraploid *Triticum turgidum* or emmer wheat (AABB genome) [10].

With annotated high-quality wheat genome sequences now available, it has become possible to design capture probe sets for wheat and to use them to accurately analyse the genome ([11]; International Wheat Genome Sequencing Consortium [IWGSC]). Sequence capture combines genotyping with *de novo* single-nucleotide polymorphism (SNP) discovery to allow allele mining and identification of rare variants. It has also been demonstrated that, using bespoke analysis tools such as CoNIFER and XHMM, copy number variants can be identified from targeted sequence capture data [12, 13]. To date, such diversity has been profiled in wheat using capture probe sets that have not been able to make use of the recent advances in wheat genome sequencing and annotation. Most of the diversity studies have implemented either complementary DNA/exon-based probe sets of 56 and 84 Mbp ( [14, 15]) or the gene-based probe set of 107 Mbp [16, 17]. Aligning the 107 Mbp capture probe set to the current wheat genome annotations (BLASTN, e-value 1e–05, identity 95%, minimum length 40 base pairs [bp]), we can see that it represents only 32.9% of the high-confidence gene set or 21.2% of the gene set plus putative promoters defined as 2 kbp upstream [11]. Similarly aligning the 84 Mbp capture probe set to the current wheat genome annotations, we can see that it represents only 32.6% of the high-confidence gene set or 20.4% of the gene set plus putative promoters. The promoter and intron sequence has previously been largely missing from capture probe sets alongside the newly characterized genes that the recent improved reference sequences have defined. There is therefore a need for an updated “gold standard” gene capture probe set for wheat, based on the current high-confidence gene models, that can be adopted by the community. High-confidence gene models have been distinguished from low-confidence models on the basis of similarity to known plant protein sequences and supporting evidence from wheat transcripts [11].

Herein, we present a gene capture probe set, which was created by integrating the current annotated wheat genome reference sequences to define a comprehensive “gold standard” gene design space for wheat. We use an island strategy, carefully spacing probes with, on average, 120 bp gaps across the design space to maximize sequencing coverage of our targets. We have also developed a comprehensive putative promoter capture probe set for wheat that takes 2 kbp upstream of the annotated genes and will facilitate global investigation to fully characterize these regulatory regions. Because approximately half of the genetic variation that is associated with phenotypic diversity in maize is found in promoter regulatory regions [18], it is reasonable to expect a similar scenario for wheat promoter regions that are poorly defined on a global scale; these are regions that need to be explored and more precisely defined across the wheat genome. The gene and putative promoter captures can be combined or used independently.

In summary, we describe 2 new wheat NimbleGen SeqCap EZ probe sets (Roche NimbleGen Inc., Madison, WI, USA), the first tiled across the genic regions of the hexaploid bread wheat genome and the second tiled across the putative promoter regions; we integrate diverse wheat material into the design to allow broad applicability of the probe sets; we validate the capture probe sets using the reference variety Chinese Spring; and we demonstrate the probe sets' application to diverse wheat accessions by enriching 8 wheat accessions that were generated by the International Maize and Wheat Improvement Center (CIMMYT), Mexico. Finally, multiplexing samples into a single capture before sequencing, using barcodes to identify individual samples in the pool, can further reduce costs; we demonstrate successful multiplexing of >20 samples in a single capture, where we can generate adequate coverage per sample for SNP calling. Our capture probe set designs are publicly available and can also be ordered directly from NimbleGen via the Roche website [19].

## Materials and Methods

### Developing the capture probe design space from its target regions

**Figure 1:**
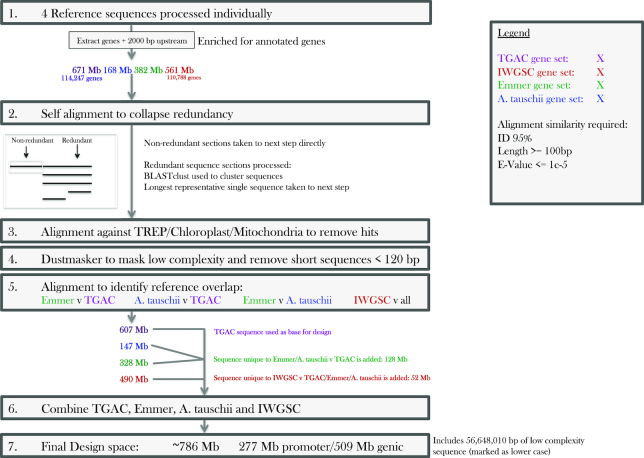
Design of the wheat gene and putative promoter capture probe sets. Processing of the TGAC Chinese Spring, IWGSC Chinese Spring, emmer, and *A. tauschii* reference sets of gene/putative promoter sequences to generate a final design space for the wheat gold standard putative promoter/gene capture probe set, i.e., NR and high complexity (Methods).

Initially the target gene/putative promoter sequences from each wheat reference genome (TGAC-Chinese Spring, IWGSC-Chinese Spring, *A. tauschii*, and emmer) were processed independently of one another. For each gene/putative promoter set (Fig. 1) gene/putative promoter sequences were aligned to themselves using BLASTN (BLASTN, RRID:SCR_001598) (version 2.2.17) with a maximum e-value of 1e–5, minimum sequence identity of 95%, and minimum match length of 100 bp. Here, nonredundant sequences with no BLASTN alignments were taken forward directly (hereafter NR sequences). Any full or partial sequences that aligned to other sequences in the gene/putative promoter set were extracted and BLASTclust was used to cluster these redundant sequences by similarity, allowing the longest representative sequence per alignment group to be identified and combined with the NR sequences to be taken forward. Furthermore, if parts of otherwise NR sequences were redundant and removed but were then outputted from the BLASTclust alignment as a representative NR sequence, these fragments were then reintegrated back into their sequence of origin. This generated a complete, reassembled where possible, set of NR sequences.

The complete set of NR sequences was aligned to the wheat chloroplast/mitochondria genomes using BLASTN, with the same parameters used previously, and regions or sequences showing hits were removed. Dustmasker (version 1.0.0) was then implemented to annotate low-complexity regions as lower case; later during probe design, probes with low-complexity regions of ≥40 bp were disregarded. Finally, NR sequences <120 bp in length were removed from the sequence set. This yielded individual probe set design spaces for TGAC wheat, IWGSC wheat, emmer, and *A. tauschii*.

These sequence sets were then compared to identify species overlap using a BLASTN alignment with the same parameters used previously. Emmer and *A. tauschii* design spaces were compared to the TGAC wheat design space and to each other, and any unique emmer/*A. tauschii* sequences were combined with the TGAC wheat design space. The Chinese Spring IWGSC design space was then compared to this TGAC/emmer/*tauschii* design space and unique sequences were combined. Finally, fragments of <75 bp in length were removed to generate a TGAC/emmer/*tauschii*/IWGSC gene and putative promoter design space.

### Exonic mature microRNA selection

Pre-microRNA (miRNA) sequences were extracted from IWGSC Refseq1.v1 using a homology-based pipeline established and optimized by Lucas and Budak [20]. These pre-miRNA sequences were mapped using BLASTN onto the chromosome sequences of the same genome to obtain their exact genomic locations. The pre-miRNA start and end alignment positions were compared to the exon boundaries of annotated genes from IWGSC and TGAC using an in-house Python script. After classification, mature miRNAs that were identified from pre-miRNAs whose start and end sites were both located in the same exonic region were directly called as exonic miRNAs. In the case of pre-miRNAs with start and end sites located on different regions, mature miRNA locations were taken into account by comparing their start and end sites with exons. If the whole mature miRNA sequence was located within an exon, they were also called as exonic miRNAs. These sequences were added to the promoter capture design space.

### Characteristics of the exome capture kit

The final gene and putative promoter capture design space (785,914,746 bp) was processed by NimbleGen (Roche NimbleGen, RRID:SCR_008571) for probe design. The NimbleGen probe set manufacturing platform has a maximum capacity of 2.16 million probes that are typically 50–100 nucleotides in length with a mean of 75 bp, i.e., maximum actual probe space ∼162 Mbp. Typically probes overlap one another to most optimally cover the target design space; however, from previous analyses we observed that a single 120 bp probe can enrich up to 500 bp routinely with adequate sequencing coverage [21]. As such, we requested that probes be tiled across our design space using an “island strategy” where probes are spaced at intervals, to most evenly cover the design space. This resulted in probes being tiled across our design space at a mean spacing of 120 bp from the 5′ start of a probe to the 5′ start of the next probe. The best probe within a 20-bp window of this start location was selected to minimize low-complexity sequence in probes and similarity to regions of the genome that were not in our target space. Low-complexity sequence had been previously marked in lower case. Similarity of probes to nontarget regions was defined using BLASTN alignment to the full wheat reference genome sequence alongside the capture design space.

### Sample library preparation and in solution captures

Genomic DNA was extracted from Chinese Spring and the 8 CIMMYT lines (21-day seedling leaf tissue) using the Qiagen DNeasy plant mini kit (Qiagen, Hilden, Germany). For Chinese Spring, 1-μg aliquots of the genomic DNA, each in a total volume of 55 μl, were sheared for 2 × 60 s using a Covaris S2 focused-ultrasonicator (duty cycle 10%, intensity 5, and 200 cycles per burst using frequency sweeping) (Covaris, Woburn, Massachusetts, US). For the 8 CIMMYT samples, 1 μg of each genomic DNA sample, in a total volume of 55 μl, was sheared for 1 × 60 s using a Covaris S2 focused-ultrasonicator (duty cycle 5%, intensity 5, and 200 cycles per burst using frequency sweeping). The fragmented DNA was directly used as input for library preparation. The NimbleGen SeqCap EZ Library SR User's Guide (Version 5.1, September 2015) was followed for all steps with the modifications listed below.

The dual size selection of the precapture libraries was adjusted to account for the larger shearing sizes. For Chinese Spring the volumes were 45 and 20 μl for right and left size selection, respectively. For the CIMMYT samples the volumes were 40 and 20 μl. Five cycles of amplification were used for the precapture polymerase chain reaction (PCR). The capture input for the Chinese Spring captures was 2 μg DNA and 1.4 μg for the CIMMYT captures. A higher input was used for Chinese Spring to increase final library yield, but it was subsequently found that 1.4 μg was sufficient. Because the input DNA was derived from wheat, 1 μl of Developer Reagent Plant Capture Enhancer (NimbleGen) was added per 100 ng input in the hybridization step instead of COT human DNA. The SeqCap HE Universal Oligo (NimbleGen) and SeqCap HE Index Oligo pool (NimbleGen) were added separately and the volume of SeqCap HE Universal Oligo was adjusted to 3.4 and 2.8 μl for the Chinese Spring and CIMMYT captures, respectively. This increase in volume was to account for the higher DNA inputs. The volume of SeqCap HE Index Oligo pool added was kept at 1 μl. Finally, for the final postcapture PCR, 14 cycles were used for the Chinese Spring captures and 12 cycles for the CIMMYT captures. The cycle number was reduced to 12 cycles because this still produced a high enough yield sequencing.

### Quality control for the putative promoter and gene capture

An initial assessment of library yield was made using Qubit High Senstivity double-stranded DNA assays (Invitrogen). Fragment size distribution was determined from Bioanalyser High Sensitivity DNA (Agilent) data. Prior to sequencing, the libraries were quantified by quantitative PCR (qPCR), using an Illumina Library Quantification Kit (KAPA) on an Applied Biosystems StepOne system.

To assist in the determination of enrichment efficiency after capture, we designed qPCR primers that cover probe targets. These are as follows for the gene capture: forward “CCGAGCCTCATAGTCAGGAG” and reverse “TGGGAAAACTGATCCCAGTC.” For the putative promoter capture probe set the recommended primers are as follows: forward “CTGTTTGTTTTGAGCGCGTC” and reverse “TGGCTTCGCGAAACTGAAAA.” The polymerase master mix from the Illumina Quantification Kit and StepOne system were used to perform the enrichment qPCR. The qPCR reaction conditions were as follows: 95°C 10 min and 40 cycles of 95°C for 10 s, 72°C for 30 s, and 60°C for 30 s. The qPCR was performed on aliquots of the capture library before and after capture, after first diluting the aliquots to the same nanograms per microliter concentration. The ∆CT between before and after successful gene capture ranged from 4 to 5. For putative promoter captures the ∆CT ranged from 3 to 4.

### Illumina DNA sequencing of gene and putative promoter capture

For the Chinese Spring sample, 4 technical replicate barcoded libraries were pooled for the gene capture and a further 4 were pooled for the putative promoter capture. The final 2 capture libraries were pooled using a ratio of 33%:66% promoter-to-gene to reflect the different size targets of the probe sets. This pool was then sequenced on a single HiSeq4000 lane. This generated 2 × 150 bp reads. For the 8 CIMMYT lines, the same barcoded libraries were used for individual gene and putative promoter captures; therefore, these captures were sequenced separately across multiple HiSeq4000 lanes. The read data produced were equivalent to 1½ and 2½ HiSeq4000 lanes for the putative promoter and gene capture, respectively.

Separate sequence capture experiments were conducted at the Kansas State University Integrated Genomics Facility using the promoter-2 capture assays following the same capture protocol with the following modifications. The capture reaction was performed on a set of 22 pooled samples barcoded using dual indexes. These samples were pooled into a larger pool of 96 barcoded sequence capture libraries and sequenced using 2 × 150 bp sequencing run on the S1 flow-cell of the NovaSeq 6000 system.

### Optimizing the capture protocol

Here the standard capture protocol described above is followed, but with the following modifications: the volume of SeqCap HE index oligo pool added to the hybridization reaction was increased to 1.2 µl for 1.4 µg input captures and the number of post-PCR amplification cycles was reduced to 10, but 8 should also yield sufficient final library for sequencing. Here, a combined gene and putative promoter probe set was used for the captures. An aliquot of the promoter-1 probe set was dried down using a vacuum concentrator at 60°C. This was then resuspended by pipetting in 2 aliquots of the gene capture probe set. The combined probe set was then divided into 2 equal-volume aliquots. Each aliquot could be used for a separate capture. The promoter-2 probe set can also be used but will result in increased enrichment of the 5′ untranslated regions (UTRs) due to probes for these regions being present in the gene and promoter-2 probe sets.

### Initial sequence data analysis

Mapping analyses of sequencing reads were carried out using BWAmem (version 0.7.10) [22] and HISAT2 (HISAT2, RRID:SCR_015530) (version 2.1.0) [23]. Paired-end reads were mapped, and only unique best mapping hits were taken forward. Mapping results were processed using SAMtools (SAMTOOLS, RRID:SCR_002105) (version 0.1.18) [24], and any nonuniquely mapping reads, unmapped reads, poor-quality reads (<Q10), and duplicate reads were removed. SNP calling was carried out using the GATK (GATK, RRID:SCR_001876) Unified genotyper (after indel realignment), which was used with a minimum quality of 50 and filtered using standard GATK recommended parameters, a minimum coverage of 5×, and only homozygous SNPs selected as defined by GATK (version 3.5.0), i.e., allele frequency in >80% of the sequencing reads [25]. Furthermore, if ≥3 SNPs occurred within a 10-bp window, these were filtered out from the calls.

## Results

### Targets of the capture probe sets

The capture probe sets target high-confidence genes and their associated putative promoters from the Chinese Spring reference genome (Fig. 1). In addition, NR gene sequences from Chinese Spring's D-genome progenitor *A. tauschii* and its AB-genome progenitor *T. turgidum* or emmer wheat were incorporated to allow broader applicability of the probe sets. For Chinese Spring–derived genes 2 genome sequence annotations were utilized: high-confidence genes from The Genome Analysis Centre (TGAC)/Earlham Institute W2RAP pipeline derived reference sequence [11], 447,729,570 bp of target sequence across 114,247 genes; and high-confidence genes from the International Wheat Genome Sequencing Consortium (IWGSC) RefSeq.v1 genome assembly, 339,580,651 bp across 110,788 genes [26]. For *A. tauschii* genes the Luo et al. [27] reference sequence was used with a high-confidence annotated gene set of 111,466,178 bp of target sequence across 28,843 genes and for emmer wheat, high-confidence genes from the Avni et al. [28] reference sequence were used, 252,137,485 bp across 65,005 genes. Only high-confidence genes were selected, and gene sequence was defined from the beginning of the 5′ UTR to the end of the 3′ UTR sequence.

Putative promoter sequence was defined in accordance with previous studies as 2000 bp upstream of the transcription start site of the aforementioned high-confidence genes as per Wicker et al. [29]. The target space for the putative promoter capture amounted to 223,409,786 bp of target sequence across 112,999 gene putative promoters and 221,681,783 bp across 110,788 gene putative promoters for the TGAC and IWGSC references, respectively. *A. tauschii*–derived putative promoters totalled 57,177,213 bp of target sequence associated with 28,843 genes, and for emmer, 130,075,005 bp associated with 65,005 genes.

In total, the combined gene and putative promoter capture target spaces amounted to 671,139,356 bp for the Chinese Spring TGAC reference, 561,262,434 bp for the IWGSC Chinese Spring reference, 168,643,391 bp for *A. tauschii*, and 382,212,490 bp for emmer (Fig. 1, Step 1). Prior to probe placement across the target space the raw gene/putative promoter sequences were processed to remove redundancy, repetitive/low-complexity sequence, and chloroplast/mitochondrial sequence (Fig. 1, Steps 2–4; Methods), resulting in probe set design spaces of 606,847,164 bp (TGAC), 490,375,105 bp (IWGSC), 328,407,758 bp (emmer), and 146,980,738 bp (*A. tauschii*). Fig. 1, Steps 5–6 show how the 606,847,164 bp of Chinese Spring TGAC sequence was used as the basis for the design and only those sequences found in emmer, *A. tauschii*, or the IWGSC sequence set that were not found with high similarity in the Chinese Spring TGAC sequence were added to the base design. As such, initially unique emmer/*A. tauschii* sequences that amounted to 127,651,054 bp were combined with the TGAC sequence. Finally, the Chinese Spring IWGSC sequence set was compared to the combined TGAC/emmer/*A. tauschii* sequences and its unique sequence space of 51,758,271 bp was also included. This ensured that gene annotation differences between the 2 main wheat references sequences were accounted for in the capture design space. As anticipated, overlap between the 2 Chinese Spring reference annotations was high.

After processing, the final combined TGAC/emmer/*tauschii*/IWGSC gene and putative promoter design space was 785,914,746 bp, of which 508,889,665 bp was gene and 277,025,081 bp was putative promoter sequence (Fig. 1, Step 7). The putative promoter design space included additional miRNA sequence totalling 900 sequences (208,968 bp). N's and low-complexity space encompassed 56,648,010 bp of the final design space; this sequence was included for probe design and later used to enable ranking of more or less preferential probes.

### Probe design

The final gene/putative promoter design space of 785,914,746 bp was used for probe design. Typically probes overlap one another to most optimally cover the target design space; however, from previous analyses we observed that a single 120-bp probe can enrich up to 500 bp with adequate sequencing coverage [21]. As such, we tiled probes (mean size 75 bp) across our design space using an “island strategy,” i.e., at intervals of on average 120 bp, to most evenly cover the design space (Methods). The gene and putative promoter probe set's predicted performance metrics and designs are summarized in Table 1. Probes in solution bind to their complementary sequence within a DNA library fragment that has typically been sheared to 200–300 bp; therefore, we bioinformatically estimated design space coverage of the probe set using shearing sizes for our simulated sequencing library of 200 bp (Methods). From this analysis we anticipate upwards of 90% coverage of both the putative promoter and gene capture design spaces with these capture probe sets. Additionally, we visualized the predicted coverage of the Chinese Spring high-confidence genes/putative promoters by their corresponding design spaces; Supplementary Fig. S1a and S1b highlight that the captures are likely to provide a comprehensive coverage of their respective targets. It is also evident that the collapse of the gene design space has been more widespread, with many regions of the capture design aligning closely to >1 target region. This is less common for the putative promoter capture design sequences that are more likely to align to a single target promoter with a longer alignment. This could be indicative of genes being more likely to have shared homology between the subgenomes of wheat or within gene families compared with promoters that may be more divergent.

**Table 1:** Probe set designs and predicted performance metrics

Probe set	Design space (bp) with Ns removed	Probe space (bp)	Design space covered by probes (%)	Estimated design space coverage if 75-bp probe captures 200 bp (bp [%])
Putative promoter	277,010,676	154,920,447	55.9	249,749,794 (90.2)
Putative promoter-2	282,328,008	160,237,779	56.8	247,535,534 (87.7)
Gene	508,560,490	16,796,494	31.8	465,988,638 (91.6)

Detailing the size of the putative promoter/gene capture design space and probe space. Estimations of the percentage coverage of the design space after sequencing if each probe captures DNA sequencing library fragments of 200 bp.

### Sequencing coverage after capture of Chinese Spring

We first examined capture efficiency using the reference variety of wheat, Chinese Spring, which the majority of the capture design space was based on. We performed putative promoter and gene captures separately using Chinese Spring DNA from 21-day seedling leaf tissue and sequenced on the HiSeq4000 (Methods). Four technical replicate barcoded libraries were pooled for the gene capture and a further 4 were pooled for the putative promoter capture, and here all 4 replicates were aligned as a single pool of reads to assess coverage (426,725,926 reads from gene and 232,437,854 from the putative promoter capture). It is clear from Table 2 that, irrespective of the reference genome implemented, the majority of reads can be aligned uniquely (mean, 77.9%) with a low duplicate rate observed (mean, 4.20%). The overall alignment rate when aligning to the full wheat reference genome was 99.8%.

**Table 2:** Coverage statistics for Chinese Spring

Gene capture: Chinese Spring 426,725,926 reads										
Reference	Reference size (bp)	% Reads aligned uniquely after duplicate removal	% Reads duplicates	No. of reference contigs	No. of reference contigs mapped	Reference contigs mapped (%)	Mean depth of coverage per reference contig	bp mapped at ≥1× (% reference covered)	bp mapped at ≥5× (% reference covered)	bp mapped at ≥10× (% reference covered)
Probe design space	426,246,621	75.2	4.52	254,950	220,837	86.6	99.15	403,219,923 (94.6%)	395,727,063 (92.8%)	377,795,215 (88.6%)
TGAC gene targets	440,066,424	71.9	5.37	114,247	112,275	98.3	73.83	426,719,705 (97.0%)	419,176,417 (95.2%)	400,456,907 (91.0%)
TGAC whole genome	13,427,354,022	89.9	3.34	735,943	733,488	99.7	5.95	10,258,685,302	2,361,028,858	996,680,117
	440,066,424^a^							(97.4%)^a^	(93.8%)^a^	(87.6%)^a^
	808,769,138^b^							(90.3%)^b^	(69.4%)^b^	(58.1%)^b^
	711,198,745^c^							(93.1%)^c^	(78.2%)^c^	(68.4%)^c^
	1,345,755,884^d^							(87.0%)^d^	(58.6%)^d^	(45.7%)^d^
	219,982,922^e^							(83.0%)^e^	(42.7%)^e^	(26.1%)^e^
Putative promoter capture: Chinese Spring 232,437,854 reads										
Probe design space	232,172,120	71.9	4.32	249,698	210,176	84.2	91.39	215,230,363 (92.7%)	208,424,502 (89.8%)	194,378,612 (83.7%)
TGAC promoter targets	219,982,922	68.1	4.64	112,999	112,600	99.6	97.51	213,924,917 (97.2%)	207,575,996 (94.4%)	194,868,834 (88.6%)
TGAC whole genome	13,427,354,022	90.4	3.02	735,943	720,291	97.9	3.83	7,746,539,630	1,221,249,873	620,781,923
	219,982,922^e^							(95.4%)^e^	(87.2%)^e^	(78.2%)^e^
	625,932,059^f^							(76.3%)^f^	(48.6%)^f^	(37.7%)^f^
	440,066,424^a^							(57.8%)^a^	(23.5%)^a^	(14.0%)^a^
	401,070,091^g^							(85.7%)^g^	(64.4%)^g^	(53.5%)^g^
	1,093,175,155^h^							(72.9%)^h^	(40.1%)^h^	(28.9%)^h^
	327,780,890^i^							(86.3%)^i^	(68.1%)^i^	(57.6%)^i^
	592,416,169^j^							(80.0%)^j^	(53.2%)^j^	(41.6%)^j^

Sequencing reads from the gene and putative promoter captures were individually aligned to their respective design spaces, targets, and the full TGAC wheat genome assembly. For alignments to the probe design space, percentages are shown excluding non–Chinese Spring–based sequence. For alignments to the gene and putative promoter targets, percentages are shown using NR sequence. For alignments to the full wheat genome, metrics are shown for coverage of the following: ^a^high-confidence genes, ^b^high-confidence genes with 2000 bp upstream and downstream, ^c^high- and low-confidence genes, ^d^high- and low-confidence genes with 2000 bp upstream and downstream, ^e^high-confidence putative promoter sequences, ^f^high-confidence putative promoters with 2000 bp upstream and downstream, ^g^high- and low-confidence putative promoters, ^h^high- and low-confidence putative promoters with 2000 bp upstream and downstream, ^i^high-confidence putative promoters with 1000 bp downstream, and ^j^high- and low-confidence putative promoters with 1000 bp downstream.

First, we aligned putative promoter and gene captured reads to their respective probe design spaces to determine enrichment efficiency in general, i.e., how much of the sequencing data were likely to have been pulled down by the probes (Table 2). A total of 75.2% and 71.9% of reads align to the gene and putative promoter probe design spaces, respectively, indicating high on-target enrichment efficiency. For the gene capture probe design space, we saw 94.6% and 92.8% of the design space with coverage at 1× and ≥5×, respectively (excluding non–Chinese Spring design space from calculations). Similarly, for the putative promoter capture design space we saw 92.7% and 89.8% of the design space with coverage at 1× and ≥5×, respectively. The performance of the putative promoter and gene capture platforms exceeded our predictions of coverage of 90.2% and 91.6%. Coverage statistics for the probe design spaces were used to identify regions with excessively high coverage, defined as coverage of >10 times the mean maximum depth of coverage for a region. Only 0.17% of gene and 0.22% of putative promoter design space regions showed such high coverage and will be removed from subsequent versions of the capture probe sets.

**Figure 2:**
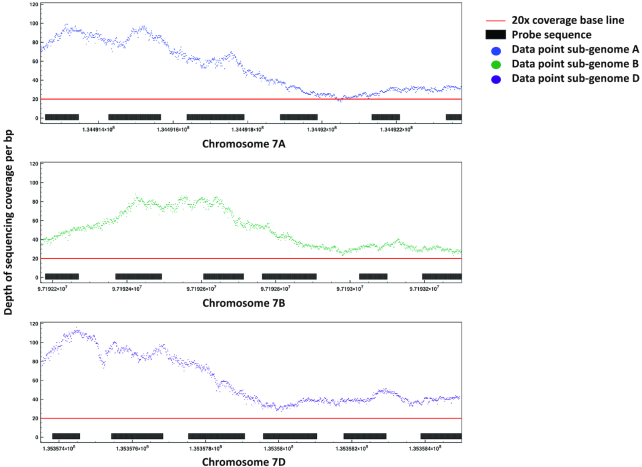
Highlighting coverage of the MYB transcription factor gene triplet using an island probe design approach. The depth of sequencing coverage is shown per base pair across 3 chromosomal intervals corresponding to a trio of homoeologous genes for the Myb transcription factor (TraesCS7A01G179900 on chr7A at 134491245–134492378 bp, TraesCS7B01G085100 on chr7B at 97192168–97193300 bp, and TraesCS7D01G181400 on chr7D at 135357355–135358494 bp).

Second, we focused on alignments to the full high-confidence gene and putative promoter spaces of Chinese Spring, i.e., only our intended targets, to determine the efficacy of our island approach and design space collapse (Table 2 and Fig. 2). For the gene capture, we observed a highly comprehensive coverage of 97.0% and 95.2% at 1× and ≥5×, respectively. Similarly, for the putative promoter capture we observed 97.2% and 94.4% coverage at 1× and ≥5×. This demonstrates exceptional performance of the island approach and Fig. 2 highlights this ability of the short probes to generate comprehensive coverage using the island approach. We noted that coverage of the full gene and putative promoter sets actually exceeds that of the probe design space. This is likely due to the full gene and putative promoter space having a smaller number of contigs (up to 114,247) that are generally longer and encompass a larger base space compared to the probe design space, which has a larger number of contigs (up to 220,837) that are shorter in length and therefore likely to hinder successful mapping of properly paired reads.

Finally, to assess off-target sequencing carryover and to ensure unbiased sequencing alignment, we looked at read alignments to the full Chinese Spring genome (Table 2). Aligning reads to the full genome reference sequence is preferential to a subset, e.g., the capture target space. This ensures correct alignment of off-target reads from sequence capture that could otherwise be incorrectly aligned to their “best fit” location in the capture target space. Here we observe coverage across 97.4% and 93.8% of the high-confidence genic regions, our targets, at 1× and ≥5×, respectively. This exceeds statistics from alignment to the design space potentially due to the inclusion of additional read pairs that traverse the transcription start site or gene end. Furthermore, we see 93.1% and 78.2% coverage at 1× and ≥5× across all high-confidence and low-confidence genes, resulting in a truly comprehensive gene capture. A total of 113,884 of the high-confidence genes (99.7%) showed sequencing coverage, with each gene covered to a mean of 97.5% at 1× and 94.5% at ≥5×. The putative promoter capture performed comparably to the gene capture, showing coverage across 95.4% and 87.2% of high-confidence putative promoters at 1× and ≥5× and also coverage of 85.7% at 1× and 64.4% at ≥5× across putative promoters associated with both high- and low-confidence genes. Here, a slightly lower coverage of low-confidence putative promoter sequences was observed than for genes, potentially due to more divergent or repetitive promoter-associated sequences. A total of 112,824 of high-confidence putative promoters (99.8%) showed sequencing coverage, with each putative promoter covered to a mean of 93.6% at 1× and 85.5% at ≥5×. Within the design space of the putative promoter capture we included miRNA sequence totalling 900 sequences (208,968 bp). We observed coverage across 92.7% of these sequences with a mean depth of 34.99× with as little as 47 million sequencing paired-end reads (23.5 million read clusters).

Using the information from the Chinese Spring sequencing validation of the gene capture probe set, we were able to develop an extended version of the putative promoter capture probe set that includes 5′ UTR sequence (Promoter-2). The 5′ UTRs for which we gained coverage of >10× across >99% of the 5′ UTR sequence were identified and up to 2 probes per 5′ UTR were added to the putative promoter capture. This resulted in the addition of 5′ UTR probes that were associated with 49,034 high-confidence genes. This provides an enhanced putative promoter capture probe set that overlaps the first probe set with the addition of the 5′ UTR.

To assess the compatibility of our capture probe set with different Chinese Spring reference genome sequences, we performed further read alignments to the full IWGSC Chinese Spring genome (RefSeqV1, Supplementary Table S1). A total of 109,862 high-confidence genes (99.2%) showed sequencing coverage, with each gene covered to a mean of 98.0% at 1× and 94.2% at ≥5×. In addition, 109,986 high-confidence putative promoters (99.3%) showed sequencing coverage, with each putative promoter covered to a mean of 90.4% at 1× and 79.0% at ≥5×, respectively. These statistics are highly comparable to the outcome using the TGAC reference and highlight the large degree of overlap that is seen between the TGAC and IWGSC Chinese Spring reference gene sets, aside from small regions of inverted duplications (Supplementary Fig. S1c). The fact that we do not see a significant difference in coverage between the TGAC and IWGSC reference sequences confirms our ability to capture much of the regions differing between the 2 references.

Using the IWGSC Chinese Spring genome that is ordered into chromosome pseudomolecules, we can visualize the genome-wide mean coverage of genes and putative promoters (Supplementary Fig. S2). We see no notable bias in coverage depth or distribution between the subgenomes of wheat or otherwise. Coverage is consistent across the vast majority of the gene and putative promoter space, with baseline means of 34.7× and 21.0× coverage. For the gene capture, the coverage coefficient of variation is 0.87, while for the putative promoter capture it is 0.79; distributions with coefficient of variation < 1 are considered low variance and as such coverage is largely uniform across the respective target spaces.

### Capturing and sequencing regions not included in the target space

Overall both captures perform well; we can typically gain >5× coverage across >90% of their intended targets and on average >20× coverage. It was noted across both captures that there was a significant proportion of low-level coverage that fell outside of high- and low-confidence genes, putative promoters, and sequences in their immediate vicinities (±2000 bp). This is visible in the ∼20% difference in reads aligning uniquely to the whole wheat genome but not to the TGAC gene/putative promoter targets. This sequence is thought to be nonenriched carryover contamination and as such could be limited by means of increased washes during the capture protocol. There is also the possibility that this may be a result of “oversequencing” of the libraries and that as such the off-target sequence will become less prominent at lower sequencing depths; however, we only see an increase in on-target sequence of 1.1% as we decrease read coverage from 440 to 100 million sequencing reads for the gene capture.

### Determination of minimum sequencing requirements

The Chinese Spring data that we have used to validate the capture probe sets originated from a single gene and a single putative promoter capture assay; however, each capture combined 4 barcoded technical replicate Chinese Spring libraries. Using different combinations of these 4 replicate libraries (all 4, 3, 2, or 1) we were able to bioinformatically reduce the number of sequencing reads in our analyses to determine the minimum sequencing requirements for coverage of the targets. Looking at the coverage of target regions with varying sequencing read numbers (Supplementary Table S2 and Supplementary Fig. S3), it is evident that increasing the number of sequencing reads increases coverage of target regions. However, there are clear saturation points for each capture probe set where further sequencing input has little to no effect on increasing target coverage. These saturation points guide our recommended sequencing levels for optimal return on investment and comprehensive coverage of capture targets at ≥5×, which is desirable for SNP calling: 200–300 million paired-end reads (100–150 million read clusters) for gene capture and 150–200 million paired-end reads for putative promoter capture (75–100 million read clusters). In Table 3 we have outlined a sliding scale of sequencing levels alongside the varying depths of coverage that they generate for the target sequences to guide user requirements (Table 3).

**Table 3:** Sequencing recommendations for gene and putative promoter capture probe sets

Capture probe set	Approximate read number required with standard protocol	Approximate read number required with optimized protocol	Expected % coverage of target (≥1×)	Expected % coverage of target (≥5×)	Expected % coverage of target (≥10×)	Mean coverage across target region^a^
Gene	100,000,000	55,000,000	94.3	69.8	35.4	9.05
Gene	200,000,000	105,000,000	96.4	86.9	68.3	17.13
Gene	300,000,000	160,000,000	97.1	91.8	81.6	25.42
Gene	400,000,000	210,000,000	97.4	93.8	87.6	34.06
Putative promoter	50,000,000	30,000,000	87.3	43.4	9.9	5.27
Putative promoter	100,000,000	55,000,000	93.2	78.2	52.6	12.05
Putative promoter	150,000,000	80,000,000	94.2	83.3	66.3	15.82
Putative promoter	200,000,000	105,000,000	95.4	87.2	78.2	21.59

Projected coverage of gene and putative promoter capture target sequence (high-confidence gene and promoter sequences, respectively) with varying numbers of sequencing reads. Also shown are the predicted read number requirements to achieve the same coverage using our optimized capture protocol (numbers rounded to the nearest 5 million reads). Read numbers are for total number of paired-end reads and should be halved to get the number of read clusters.

^a^Target region is defined as all gene or putative promoter sequences that the probe sets are tiled across, i.e., including padding between probes.

### Mulitplexing to generate comprehensive coverage

**Figure 3:**
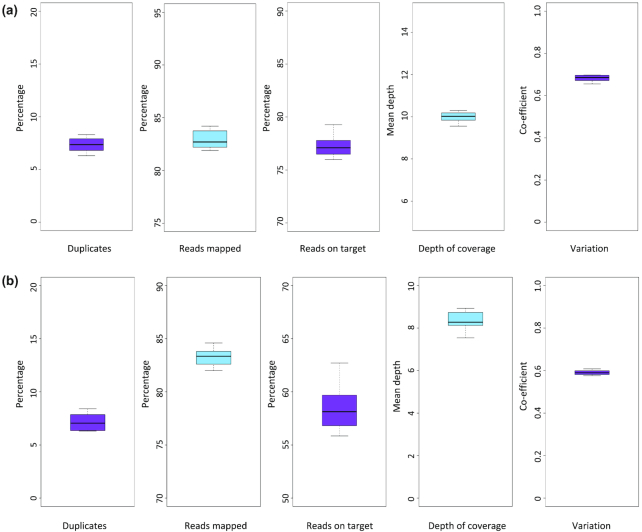
Summary statistics for the 8-plex gene and promoter capture tests. We performed read alignments for the 8 CIMMYT samples to the full Chinese Spring genome. For the **(a)** gene capture and **(b)** putative promoter capture probe sets, from left to right, we show box and whisker plots for the percentage of sequencing reads per sample that were identified as duplicates, the percentage of reads mapping uniquely to the whole genome reference sequence, the percentage of reads defined as “on target,” i.e., aligned to the capture probe design space, the mean depth of coverage per sample, and the coefficient of variation per sample.

Multiplexing DNA from multiple wheat lines and enriching them in a single capture reaction before sequencing can decrease costs. It is important to determine whether such a large capture probe set with the “island strategy” probe design will yield uniform coverage of multiple samples. First, we multiplexed 8 different samples per gene and putative promoter capture to compare performance metrics with our previous single sample capture. We used 8 diverse wheat accessions that were generated by CIMMYT, Mexico. Importantly, these accessions are unrelated to the reference variety landrace Chinese Spring that we used for probe design. Furthermore, these accessions are products of complex breeding programmes and represent both elite and exotic material [30] (Supplementary Table S3). We sequenced the gene capture multiplexed pool to a depth of 800 million paired-end reads (∼100 million paired-end reads per sample or 50 million read clusters) and the putative promoter capture pool to 600 million paired-end reads (∼75 million reads per sample or 37.5 million read clusters). We performed read alignments for the 8 samples to the full Chinese Spring genome (Supplementary Tables S4 and S5). Uniform and successful enrichment of the 8 samples was observed with both the gene and putative promoter captures. All samples show a high percentage of reads aligned on target (77.25% and 58.47% on average for the gene and putative promoter captures, respectively) with low variation between samples represented by interquartile ranges of <5% (Fig. 3). Coefficients of variation for the gene and putative promoter capture were 0.68 and 0.59, respectively; these values are considered low variance, and, as such, coverage is largely uniform across the respective target spaces. All samples covered the gene target regions at ≥5× to between 69.2% and 73.1% and the putative promoter target regions at ≥5× to between 62.7% and 70.4% (Supplementary Tables S4 and S5). For each of the samples, coverage of target regions was higher than the expected coverage that was predicted on the basis of the depth of sequencing from the Chinese Spring enrichment (Supplementary Fig. S4).

Second, we validated our promoter-2 capture probe set that includes 5′ UTR sequence whilst also multiplexing a larger number of samples for capture (22 samples). For this analysis we used a diverse set of wheat lines that were selected on the basis of genotyping with the 9K iSelect array [31]. We sequenced the promoter-2 capture multiplexed pool to a mean depth of ∼46 million paired-end reads per sample (23 million read clusters) and aligned on average 85.5% of reads uniquely to the reference genome. Across a representative subset of the samples, the mean depth of coverage for the putative promoter high-confidence target regions ranged from 6.2× to 6.9× with 13.5–17.1% of this space covered at ≥10×. These metrics surpass our expected depth of coverage on target using 50 million paired-end reads, for which we predicted a mean coverage of 5.3× and 9.9% coverage at ≥10× (Table 3). We also noted low variation between samples, represented by interquartile ranges of *<*5% for coverage of the targets at ≥1×, 5×, and 10×. This analysis demonstrates uniform successful enrichment of the samples and that multiplexing >20 samples for capture had no detrimental effect.

### Genotyping sensitivity of the capture probe sets

We focused on our 8-plex test, in which we sequenced the samples to our recommended sequencing depth for SNP calling, and we identified homozygous SNPs in each of the samples at positions where we saw ≥5× coverage (Methods). On average the samples had 1,031,677 SNPs each from the gene capture and 968,640 SNPs each from the putative promoter capture. Furthermore, when we focused on locations where each of the 8 samples either had a SNP identified or else had ≥5× coverage with no SNP, i.e., the reference allele, this resulted in 1,019,556 positions that were available for comparison across the sample set for the gene capture and 869,954 for the putative promoter capture. This highlights our ability to perform *de novo* SNP discovery with captured sequencing data, the high level of diversity in the 8 CIMMYT lines compared with the Chinese Spring reference, and the successful uniform enrichment of these samples despite this diversity.

### Optimizing the capture protocol

Owing to the large size of our capture probe sets we performed further optimization of the standard NimbleGen capture protocol to focus our sequencing reads on target as much as possible. We again used Chinese Spring for this analysis in a repeat of our initial quality control of the capture. Here, we combined both the putative promoter and gene capture probe sets for analysis and increased the volume of indexed blocking oligonucleotides used per capture (Methods). For this analysis we noted that 57% of the mapped reads were on target, i.e., aligned directly to the probe design. This is in line with what we observed previously, with a range of 58.47–77.25% observed across the gene and putative promoter captures. However, we noted that here, rather than enriching high- and low-confidence genes, there was a bias specifically towards high-confidence genes, with a 1.9-fold increase in the sequence space aligning to these genes compared with previous analyses. This allowed us to lower our original predictions of sequencing requirements for adequate coverage of the high-confidence gene set (Table 3).

## Discussion

Sequence capture is rapidly becoming one of the main techniques employed by the wheat research community for resequencing of the large complex wheat genome at reduced cost. It allows the identification of previously uncharacterized genetic variation in the form of SNPs and indels in key regions of interest that are typically gene associated. To date, many studies have implemented either exon or complementary DNA–based capture probes sets that have not been able to make use of the recent advances in wheat genome sequencing and annotation. Furthermore, promoter and intronic sequence has largely been missing from capture probe sets. Herein, we present and validate a gene capture probe set, created by integrating the current annotated wheat genome reference sequences to define a comprehensive “gold standard” gene design space for bread wheat. We have also developed a comprehensive putative promoter capture probe set for wheat that covers 2 kbp upstream of the annotated genes and will facilitate global investigation to fully characterize these regulatory regions. An updated version of the putative promoter capture probe set also includes gene 5′ UTRs and so will capture regulatory elements within these regions.

We have demonstrated the use of the capture probe sets to analyse a diverse set of material including pure breeding lines that were generated by CIMMYT, Mexico. We studied the consistency of our data by correlating the sequence coverage depths between independent captures for multiple DNA samples. In addition, we successfully multiplexed >20 samples in a single capture with no dropout of capture efficiency despite the large size of our capture. From multiplexed captures, we can generate adequate coverage per sample for SNP calling, resulting in a lower cost per sample for gene and putative promoter captures. This brings down the cost of resequencing the entirety of wheat's gene-associated space. Furthermore, it is likely that, because no reduced capture efficiency was observed with a 20-plex capture, more samples could be multiplexed without a detrimental effect. We have focused on generating a depth of coverage that is adequate for SNP calling, but the potential is there for skim sequencing samples. Skim sequencing generates low coverage for a larger number of lines to allow allele mining at reduced cost, and this can be achieved using multiplexing or bulk segregant analysis, which we have previously combined successfully with wheat exome capture [16].

This assay brings resequencing of the entirety of the high-confidence gene-associated portion of wheat within the reach of the wheat community. Our multiplexing analysis defined >1.8 million positions across 8 diverse samples, in which each of the samples had ≥5× coverage to allow comparison, and variation was observed between samples. This level of SNP information will allow refinement of key genetic regions linked to traits and enable researchers to pinpoint phenotype-inducing SNPs more precisely. Current methods such as genotyping by sequencing typically yield far fewer usable SNPs, with <20,000 reported [32, 33]. In the case of SNP arrays, the largest commonly reported array for wheat is 819,571 SNPs, although previous analyses reported only a small proportion of these SNPs to be polymorphic in analysed accessions (112,723 in a diverse panel similar to that used here) and no indels or rearrangements can be profiled using this methodology [34]. Finally, we predict that our optimization of the protocol for this large-scale capture using an island approach will allow us to sequence >90% of the gene space of up to 4 wheat accessions on a single HiSeq4000 lane and 20 accessions on a NovaSeq S1 flow cell to ≥5× (>80% at >10×). Our capture probe set design is publicly available and can also be ordered directly from NimbleGen via the Roche website [19].

## Conclusion

We have previously demonstrated the use of sequence capture to allow the study of both genotype and DNA methylation across targeted regions in wheat [21]. Using bisulfite treatment after sequence capture, DNA methylation analyses can be performed using the same probe sets that are implemented for genotyping [35].

Moreover, we have demonstrated the use of sequence capture that was designed using the reference wheat variety Chinese Spring to analyse diverse landraces from the Watkins collection [36] and even highly divergent ancient wheat diploid progenitors with high efficiency [37, 38]. As such, it is likely that the capture probe sets defined here not only could effectively enable resequencing of the high-confidence genes of bread wheat lines, but they could be used to further epigenetics research and research across a broader variety of wheat accessions than we tested here. The integration of more diverse wheat diploid and tetraploid progenitor material into the design will also allow broad applicability of the probe sets to varieties beyond bread wheat and also to the synthetic wheat lines, constructed from diploid and tetraploid progenitors, that are becoming increasingly popular in the wheat community.

## Availability of supporting data and materials

The sequencing data sets supporting the results of this article are available in the European Nucleotide Archive repository, study accession number PRJEB27620 and sample accession numbers SAMEA4777554-577. The final design space for the capture probes sets and the locations of the capture probes on this design space are available from the Grassroots Data Repository [39]. The target locations of the capture probe sets on the Chinese Spring IWGSC RefSeqv1, i.e., the high-confidence gene and putative promoter sequences, are detailed in supporting files 2, 3, and 4. The NimbleGen order numbers for the probe sets are as follows: Gene Capture 4 000 026 820; Promoter Capture v1, 4 000 030 160; and Promoter Capture v2, 4 000 032 530. All data are also available from the *GigaScience* GigaDB repository [40].

## Additional files

File 1: Supplementary_data.docx includes Supplementary Figures S1-S4, Supplementary Tables S1-S5.

File 2: Gene-capture-HC-targets.bed.

File 3: Prom-capture-HC-targets.bed.

File 4: Prom-capture-HC+5UTR-targets.bed.

## Abbreviations

bp, base pairs; CIMMYT: International Maize and Wheat Improvement Center (Centro Internacional de Mejoramiento de Maíz y Trigo); IWGSC: International Wheat Genome Sequencing Consortium; miRNA: microRNA; NR: nonredundant; PCR: polymerase chain reaction; qPCR: quantitative polymerase chain reaction; SNP: single-nucleotide polymorphism; TGAC: The Genome Analysis Centre (now known as the Earlham Institute); UTR: untranslated region.

## Consent for publication

All plants used in this study were grown in controlled growth chambers complying with Norwich Research Park guidelines.

## Competing interests

The authors declare that they have no competing interests.

## Funding

This project was supported by the Biotechnology and Biological Sciences Research Council via ERA-CAPS grant BB/N005104/1, BB/N005155/1 (L.G., A.H.), and BBSRC Designing Future Wheat BB/P016855/1 (A.H.). Sequencing of the CIMMYT accessions was supported by BBS/OS/NW/000017 (T.B.). US group efforts were supported by National Research Initiative Competitive grants 2017-67007-25939 (Wheat-CAP) and 2016-67013-24473 from the US Department of Agriculture National Institute of Food and Agriculture.

## Author contributions

The capture probe set design, Chinese Spring, and CIMMYT line bioinformatic validation and manuscript preparation were performed by L-J.G. The project was designed, planned, and conducted by L-J.G. and A.H. Plant growth, DNA extractions, library preparation, and sequence capture were performed by T.B. with assistance from L.C. T.R. assisted with capture probe design. The CIMMYT material was contributed by S.S. E.A. and H.B. contributed to the promoter capture assay design. A.A. conducted sequence capture and NGS sequencing. K.J. contributed to analysing promoter capture data for the 22-plex test. All authors read and approved the final manuscript.

## Supplementary Material

GIGA-D-18-00253_Original_Submission.pdf

GIGA-D-18-00253_Revision_1.pdf

GIGA-D-18-00253_Revision_2.pdf

Response_to_Reviewer_Comments_Original_Submission.pdf

Response_to_Reviewer_Comments_Revision_1.pdf

Reviewer_1_Report_Original_Submission -- Dongying Gao 10/24/2018 Reviewed

Reviewer_1_Report_Revision_1 -- Dongying Gao 12/6/2018 Reviewed

Reviewer_2_Report_Original_Submission -- Robert van Buren 10/26/2018 Reviewed

Supplemental Files
