# Supplementary material for: Integrating genomic resources to present full gene and putative promoter capture probe sets for bread wheat
Source: Gigascience. 2019 Jan 31;8(4):giz018. doi: 10.1093/gigascience/giz018 (PMC6461119; doi:10.1093/gigascience/giz018)
Supplement: Supplemental Files [file giz018_supplemental_files.zip › Supplementary_data-new.pdf]

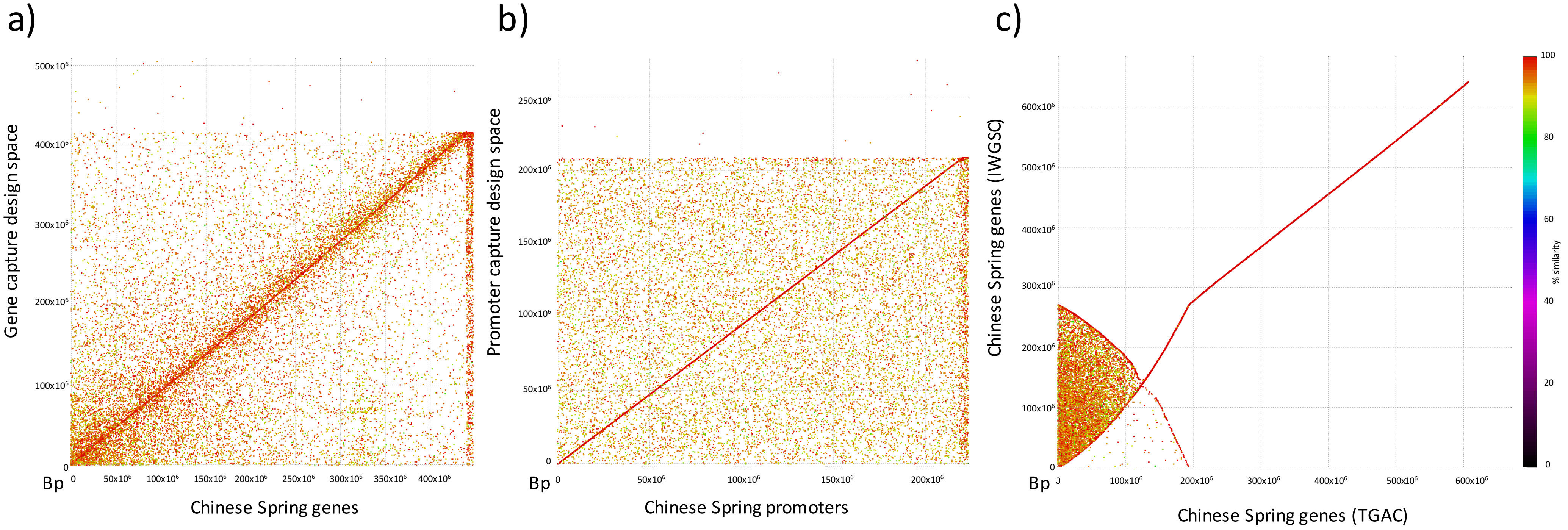

Figure S1. Aligning reference sequences for visualization. Using the software mummerplot we produced an alignment dotplot where a sequence is laid out on each axis and a point is plotted at every position where the two sequences show similarity. The % similarity denotes the percentage similarity between two individual sequences. Within each plot all sequences are ordered and orientated such that the largest hits cluster near the main diagonal. **(a)** Using the Chinese spring TGAC high confidence gene set as the reference and the gene capture design space as the query with all alignments reported **(b)** Using the Chinese spring TGAC high confidence promoter set as the reference and the promoter capture design space as the query with all alignments reported **(c)** Using the Chinese spring TGAC high confidence gene set as the reference and the Chinese Spring IWGSC high confidence gene set as a query. Here we map each position in the query sequence to its best hit in the reference, allowing for reference overlaps.

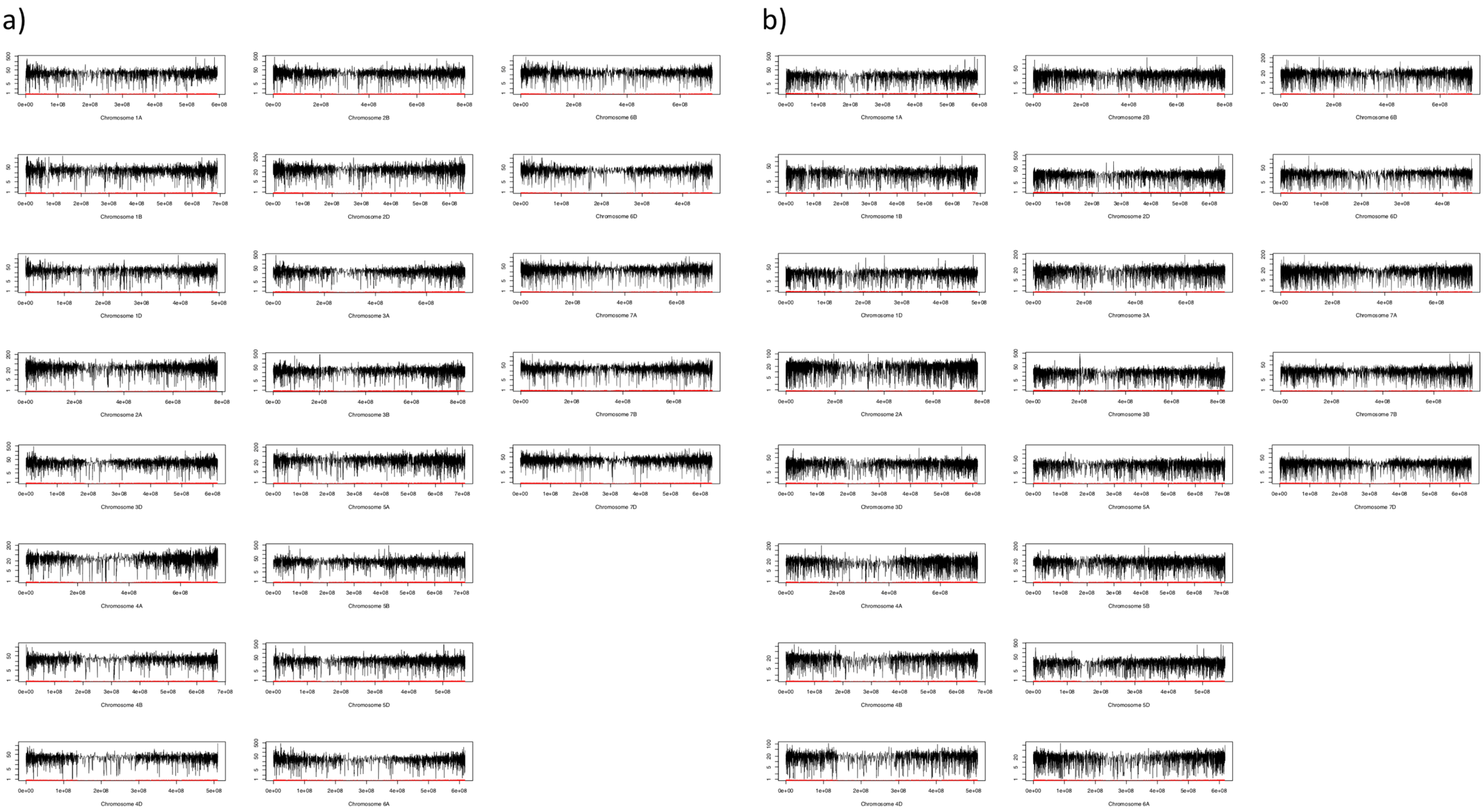

Figure S2. Visualizing coverage across the gene and promoter space of Chinese Spring wheat. Using the IWGSC Chinese Spring reference sequence, the average coverage per target region was calculated and is shown here as a line plot for each chromosome with scores assigned to the start position of the target region. The y-axis uses a log-scale to show the average depth of sequencing coverage per region. The x-axis shows the position in bp along each chromosome. Vertical red bars along the x-axis depict the start sites of all target regions, independently of whether they show sequencing coverage or not. **(a)** Using the Chinese spring IWGSC high confidence gene set as the reference **(b)** Using the Chinese spring IWGSC high confidence promoter set as the reference.

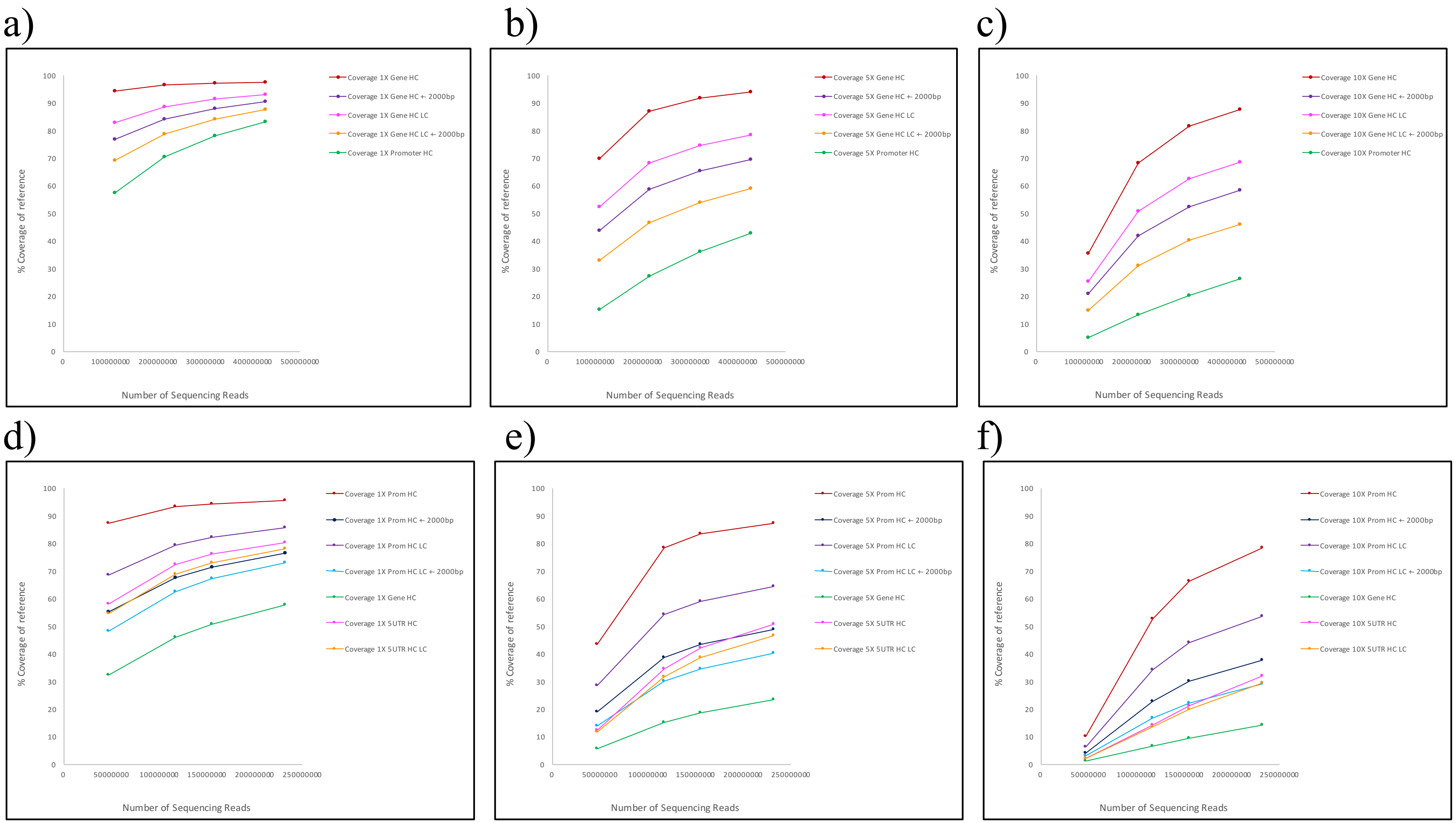

Figure S3. Summary of sequencing data from the gene and promoter capture probe set. The effect of increasing the number of sequencing reads on the percentage coverage of the individual reference sequences for; high confidence genes (Gene HC), high confidence genes with 2000bp upstream and 2000bp downstream (Gene HC+- 2000bp), high and low confidence genes (Gene HC LC), high and low confidence genes with 2000bp upstream and 2000bp downstream (Gene HC LC +- 2000bp), high confidence promoter sequences (Promoter HC), high confidence promoters with 2000bp upstream and 2000bp downstream (Prom HC+- 2000bp), high and low confidence promoters (Prom HC LC), high and low confidence promoters with 2000bp upstream and 2000bp downstream (Prom HC LC +- 2000bp), high confidence promoters with 1000bp downstream (Prom HC + 1000bp DS) and high and low confidence promoters with 1000bp downstream (Prom HC LC + 1000bp DS). **(a)** shows the percentage of each of the references sequences covered at >= 1X after gene capture, **(b)** shows the percentage of each of the references sequences covered at >= 5X after gene capture, **(c)** shows the percentage of each of the references sequences covered at >= 10X after gene capture **(c)** shows the percentage of each of the references sequences covered at >= 1X after promoter capture, **(b)** shows the percentage of each of the references sequences covered at >= 5X after promoter capture, **(c)** shows the percentage of each of the references sequences covered at >= 10X after promoter capture**.**

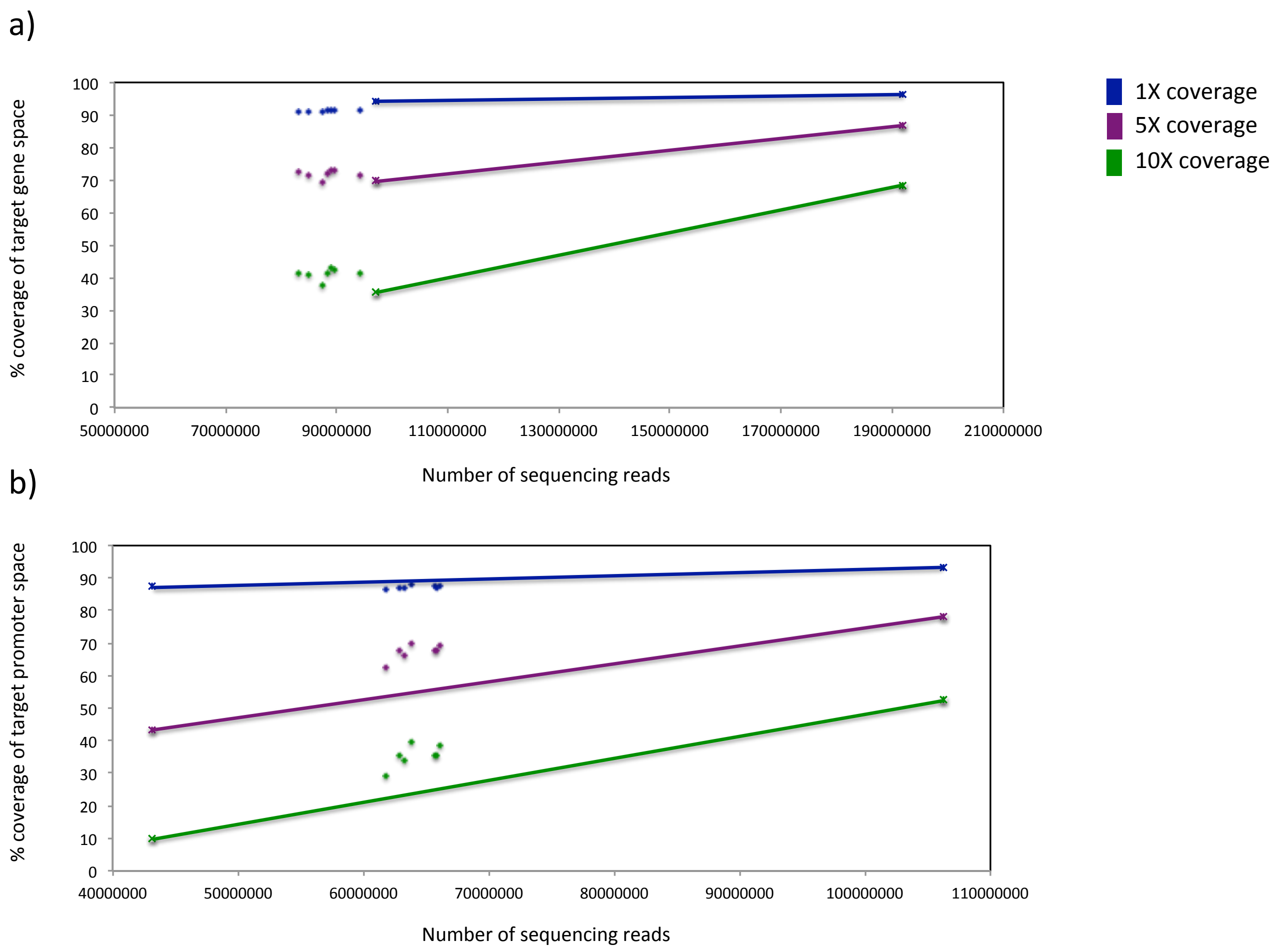

Figure S4. Summary of sequencing data for the 8 multiplexed samples after gene and promoter capture probe set. Here we show the percentage coverage of the target gene and promoter space by each of the 8 samples that were captured using the respective capture probe sets. The percentage reference coverage is reported with respect to the number of sequencing reads generated per sample and colour coded dependent on the minimum coverage depth. For comparison the same information is reported for the closest comparative number of sequencing reads that were generated for Chinese Spring sample that was taken through the gene and promoter captures as a 1-plex, data points for this sample are denoted with an X and joined using a solid line.

Table S1. Coverage statistics for Chinese Spring. Sequencing reads from the gene and promoter captures were individually aligned to the IWGSC RefSeq.v1 full wheat genome assembly. Metrics are shown for coverage of; high confidence genes (Gene HC), high confidence genes with 2000bp upstream and downstream (Gene HC+- 2000bp), high and low confidence genes (Gene HC LC), high and low confidence genes with 2000bp upstream and downstream (Gene HC LC +- 2000bp), high confidence promoter sequences (Prom HC), high confidence promoters with 2000bp upstream and downstream (Prom HC+- 2000bp), high and low confidence promoters (Prom HC LC), high and low confidence promoters with 2000bp upstream and downstream (Prom HC LC +- 2000bp).

**Gene capture: Chinese Spring 426,725,926 Reads**												
**Reference**	**Reference size<br>-Non-Redundant<br>-Chinese Spring Only**	**Reads aligned**	**Reads aligned uniquely**	**Reads uniquely mapped post remove duplicates**	**% Reads uniquely mapped post remove duplicates**	**% Reads duplicates**	**Ref contig number**	**Average % ref contigs mapped**	**Average depth of cov per ref contig**	**Bp mapped (1X)**	**Bp mapped (5X)**	**Bp mapped (10X)**
RefSeq.v1 Whole genome	14,547,261,565<br>Gene HC: 338,512,307<br>Gene LC HC: 543,067,965	425,575,663	394,213,256	381,199,959	89.3	3.06	22	74.13	4.90	11,158,532,693<br>328,098,412:96.9%<br>501,229,705:92.3%	2,331,691,653<br>313,163,614:92.5%<br>418,896,653:77.1%	978,154,857<br>293,842,619:86.8%<br>374,269,766:68.9%
**Promoter capture: Chinese Spring 232,437,854 Reads**												
**Reference**	**Reference size<br>-Non-Redundant<br>-Chinese Spring Only**	**Reads aligned**	**Reads align uniquely**	**Reads align uniquely post remove duplicates**	**%Reads align uniquely post remove duplicates**	**% Reads duplicates**	**Ref contig number**	**Average % ref contigs mapped**	**Average depth of cov per ref contig**	**Bp mapped (1X)**	**Bp mapped (5X)**	**Bp mapped (10X)**
RefSeq.v1 Whole genome	14,547,261,565<br>Prom HC: 217,920,426<br>Prom HC LC: 486,428,496	231,812,551	215,057,555	208,620,842	89.8	2.78	22	55.89	3.55	8,343,232,608<br>198,975,936:91.3%<br>389,219,924:80.0%	1,200,947,923<br>173,959,400:79.8%<br>267,154,488:54.9%	612,161,162<br>152,052,088:69.8%<br>211,322,973:43.4%

Table S2. Coverage statistics for four barcoded Chinese Spring libraries. Sequencing reads from gene and promoter captures were individually aligned to the full wheat genome assembly for; all Chinese Spring sequencing reads (four barcoded libraries), three combined barcoded libraries, two combined barcoded libraries and a single barcoded library. Metrics are shown for coverage of; high confidence genes (Gene HC), high confidence genes with 2000bp upstream and downstream (Gene HC+- 2000bp), high and low confidence genes (Gene HC LC), high and low confidence genes with 2000bp upstream and downstream (Gene HC LC +- 2000bp), high confidence promoter sequences (Prom HC), high confidence promoters with 2000bp upstream and downstream (Prom HC+- 2000bp), high and low confidence promoters (Prom HC LC), high and low confidence promoters with 2000bp upstream and downstream (Prom HC LC +- 2000bp).

Reference size: 13,427,354,022 Gene HC: 440,066,424 Gene HC +-2000: 808,769,138 Gene LC HC: 711,198,745 Gene LCHC+-2000:1,345,755,884

Chinese Spring gene capture library	Reads	Reads aligned	Reads aligned uniquely	Reads uniquely mapped post remove duplicates	% Reads uniquely mapped post remove duplicates	% ref contigs mapped	Average depth of cov per ref contig	Bp mapped (1X)	Bp mapped (5X)	Bp mapped (10X)
4 libraries	426,725,926	425,695,014	396,993,123	383,731,064	89.9	99.67	5.95	10,258,685,302<br>428,735,096:97.4%<br>730,001,835:90.5%<br>661,936,335:93.1%<br>1,171,303,906:87.6%	2,361,028,858<br>412,762,488:93.8%<br>561,007,567:69.6%<br>556,006,951:78.2%<br>788,292,343:59.0%	996,680,117<br>385,437,438:87.6%<br>470,086,102:58.3%<br>486,715,016:68.4%<br>615,106,357:46.0%
3 libraries	319,640,574	318,989,580	297,395,790	287,570,064	90.0	99.29	4.77	9,265,075,217<br>427,350,883:97.1%<br>709,639,526:88.0%<br>649,665,883:91.3%<br>1,124,689,762:84.1%	1,719,058,051<br>403,943,548:91.8%<br>526,470,023:65.3%<br>530,839,434:74.6%<br>720,580,166:53.9%	801,322,341<br>358,985,928:81.6%<br>421,799,332:52.3%<br>442,739,772:62.3%<br>537,960,994:40.2%
2 libraries	213,532,272	213,106,658	198,642,795	191,983,278	89.9	98.21	3.60	7,735,665,004<br>424,418,352:96.4%<br>677,416,465:84.0%<br>629,334,908:88.5%<br>1,051,151,967:78.6%	1,153,705,315<br>382,271,864:86.9%<br>471,773,929:58.5%<br>485,788,087:68.3%<br>622,761,163:46.6%	566,099,281<br>300,602,495:68.3%<br>338,105,111:41.9%<br>359,557,778:50.6%<br>416,296,932:31.1%
1 library	108,382,748	108,084,311	100,807,293	97,168,623	89.7	93.50	2.39	5,303,184,461<br>415,159,243:94.3%<br>619,135,837:76.8%<br>588,827,333:82.8%<br>921,700,372:69.0%	626,705,276<br>306,985,675:69.8%<br>351,475,199:43.6%<br>370,897,365:52.2%<br>437,985,072:32.8%	243,032,065<br>155,898,041:35.4%<br>167,392,407:20.8%<br>179,984,099:25.3%<br>197,276,776:14.8%

Reference size: 13,427,354,022 Prom HC: 219,982,922 Prom HC +- 2000: 625,932,059 Prom HC LC: 401,070,091 Prom HC LC +-2000:1,093,175,155

Chinese Spring promoter capture library	Reads	Reads aligned	Reads align uniquely	Reads align uniquely post remove duplicates	%Reads align uniquely post remove duplicates	% ref contigs mapped	Average depth of cov per ref contig	Bp mapped (1X)	Bp mapped (5X)	Bp mapped (10X)
4 libraries	232,437,854	231,901,783	216,749,581	210,203,439	90.4	97.87	3.83	7,746,539,630<br>209,758,402:95.4%<br>477,892,749:76.4%<br>343,746,168:85.7%<br>796,753,146:73.1%	1,221,249,873<br>191,925,388:87.2%<br>304,234,069:48.7%<br>258,090,704:64.4%<br>437,947,942:40.2%	620,781,923<br>172,033,599:78.2%<br>235,687,639:37.7%<br>214,466,113:53.5%<br>316,068,537:29.0%
3 libraries	155,483,978	155,078,260	145,017,502	141,098,014	90.7	95.49	2.95	6,294,568,291<br>207,261,077:94.2%<br>446,922,142:71.5%<br>329,229,493:82.1%<br>731,033,326:67.1%	864,207,837<br>183,217,056:83.3%<br>271,182,719:43.4%<br>237,150,625:59.1%<br>376,801,487:34.6%	427,008,585<br>145,948,659:66.3%<br>187,210,947:29.9%<br>176,048,795:43.9%<br>242,182,293:22.2%
2 libraries	117,086,184	116,808,960	109,229,583	106,304,879	90.8	93.1	2.54	5,335,121,601<br>204,936,344:93.2%<br>422,900,659:67.6%<br>317,477,606:79.2%<br>680,923,116:62.5%	683,552,372<br>171,982,330:78.2%<br>241,818,895:38.7%<br>216,957,631:54.1%<br>327,909,098:30.1%	300,776,158<br>115,669,028:52.6%<br>142,810,928:22.8%<br>136,981,040:34.2%<br>180,880,742:16.6%
1 library	47,454,980	47,332,140	44,244,510	43,188,702	91.0	80.09	1.76	2,974,023,846<br>192,138,485:87.3%<br>345,700,131:55.3%<br>275,243,975:68.6%<br>526,833,311:48.3%	256,133,492<br>95,540,448:43.4%<br>118,965,714:19.0%<br>113,516,221:28.3%<br>151,326,987:13.9%	48,668,669<br>21,784,978:9.9%<br>25,425,302:4.1%<br>25,197,051:6.3%<br>31,345,350:2.9%

Table S3. Details of the 8 CIMMYT wheat accessions that were used for multiplexing.

Sample	Cross	Details
5	WBLL1/KUKUNA//TACUPETO F2001/3/BAJ #1	Early maturity, high yield, heat tolerant
8	HUW234+LR34/PRINIA*2//KIRITATI	Early maturity, high yield, heat tolerant, Ug99 stem rust resistant
11	CNDO/R143//ENTE/MEXI_2/3/AEGILOPS SQUARROSA (TAUS)/4/WEAVER/5/PICUS/6/TROST/7/TACUPETO F2001	High yield
15	NELOKI	High yield
17	BORLAUG100 F2014	Released in Mexico and Bolivia, high yield in various environments, good bread quality
19	KACHU #1/KIRITATI//KACHU	Earlier maturity, high yield, APR to Ug99 stem rust
23	VOROBEY	Drought and heat tolerant, resistant to Septoria tritici blotch
24	ROELFS F2007	Released in Mexico, high yield and drought tolerance, good bread quality

Table S4. Coverage statistics for 8 multiplexed CIMMYT libraries after gene capture. Sequencing reads from the gene capture were individually aligned to the full wheat genome assembly. Metrics are shown for coverage of; high confidence genes (Gene HC) and high and low confidence genes (Gene HC LC).

Reference size: 13,427,354,022 Gene HC: 440,066,424 Gene LC HC: 711,198,745

Sample	Reads	Reads aligned	% Reads align 1st pass	Reads aligned uniquely	Reads uniquely mapped post remove duplicates	% Reads uniquely mapped post remove duplicate	% Reads duplicate	Bp mapped (1X)	Bp mapped (5X)	Bp mapped (10X)	Homozygous SNP Number (min 5X coverage)
CS-1	108,382,748	108,084,311	99.7	100,807,293 (93.0%)	97,168,623	89.7	3.3	5,303,184,461<br>415,159,243:94.3%<br>588,827,333:82.8%	626,705,276<br>306,985,675:69.8%<br>370,897,365:52.2%	243,032,065<br>155,898,041:35.4%<br>179,984,099:25.3%	26,093
5	105,864,308	104,776,945	99.0	95,725,397 (90.4%)	88,505,431	83.6	6.8	4,581,790,221<br>402,266,248:91.4%<br>568,043,863:79.9%	639,625,456<br>316,059,080:71.8%<br>383,027,670:53.9%	280,510,458<br>182,123,496:41.4%<br>209,989,146:29.5%	1,106,688
8	111,888,106	111,024,531	99.2	101,164,129 (90.4%)	94,159,750	84.2	6.3	4,786,532,653<br>401,858,200:91.3%<br>567,618,130:79.8%	668,258,379<br>314,854,697:71.5%<br>382,757,514:53.8%	289,589,803<br>181,995,136:41.4%<br>210,696,848:29.6%	1,112,307
11	111,473,814	110,602,362	99.2	101,084,272 (90.7%)	93,522,592	83.9	6.8	4,784,278,968<br>402,667,220:91.5%<br>569,824,125:80.1%	667,070,083<br>319,606,759:72.6%<br>388,527,556:54.6%	295,381,154<br>187,690,913:42.7%<br>217,163,027:30.5%	1,081,946
15	109,371,280	108,601,717	99.3	98,693,170 (90.2%)	89,602,935	81.9	8.3	4,800,121,863<br>402,707,900:91.5%<br>565,762,474:79.6%	619,178,430<br>321,786,223:73.1%<br>385,913,204:54.3%	276,148,407<br>186,024,106:42.3%<br>212,463,046:29.9%	966,594
17	107,684,170	106,722,271	99.1	96,839,795 (89.9%)	88,860,503	82.5	7.4	4,749,852,127<br>402,549,212:91.5%<br>565,214,047:79.5%	610,468,523<br>320,815,759:72.9%<br>384,342,980:54.0%	275,848,160<br>187,777,229:42.7%<br>214,180,548:30.1%	984,249
19	100,759,196	99,373,531	98.6	90,491,876 (89.8%)	83,135,722	82.5	7.3	4,470,962,771<br>401,512,886:91.2%<br>561,836,769:79.0%	587,475,854<br>319,559,061:72.6%<br>381,124,493:53.6%	262,091,770<br>183,117,085:41.6%<br>207,849,053:29.2%	966,523
23	103,694,962	102,738,043	99.1	93,335,309 (90.0%)	84,960,355	81.9	8.1	4,618,280,860<br>401,036,244:91.1%<br>561,208,452:78.9%	590,472,456<br>315,352,492:71.7%<br>376,791,566:53.0%	261,951,999<br>180,191,125:40.9%<br>205,133,130:28.8%	1,081,302
24	105,481,540	105,042,211	99.6	95,548,582 (90.6%)	87,428,338	82.9	7.7	4,761,737,913<br>399,806,897:90.9%<br>557,952,117:78.5%	596,452,546<br>304,480,206:69.2%<br>364,606,767:51.3%	251,334,623<br>166,120,433:37.7%<br>190,434,543:26.8%	953,809

Table S5. Coverage statistics for 8 multiplexed CIMMYT libraries after promoter capture. Sequencing reads from the promoter capture were individually aligned to the full wheat genome assembly. Metrics are shown for coverage of; high confidence promoter sequences (Prom HC), high and low confidence promoters (Prom HC LC).

Reference size: 13,427,354,022 Prom HC: 219,982,922 Prom HC LC: 401,070,091

Sample	Reads	Reads aligned	% Reads align 1st pass	Reads align uniquely	Reads align uniquely post remove duplicates	%Reads align uniquely post remove duplicate	% Reads duplicate	Bp mapped (1X)	Bp mapped (5X)	Bp mapped (10X)	Homozygous SNP Number (min 5X coverage)
CS-1	47,454,980	47,332,140	99.7	44,244,510 (93.2%)	43,188,702	91.0	2.2	2,974,023,846<br>192,138,485:87.3%<br>275,243,975:68.6%	256,133,492<br>95,540,448:43.4%<br>113,516,221:28.3%	48,668,669<br>21,784,978:9.9%<br>25,197,051:6.3%	3,170
CS-2	117,086,184	116,808,960	99.8	109,229,583 (93.3%)	106,304,879	90.8	2.5	5,335,121,601<br>204,936,344:93.2%<br>317,477,606:79.2%	683,552,372<br>171,982,330:78.2%<br>216,957,631:54.1%	300,776,158<br>115,669,028:52.6%<br>136,981,040:34.2%	7,656
5	77,944,570	77,174,617	99.0	70,361,170 (90.3%)	63,823,301	82.0	8.4	3,694,172,819<br>193,092,334:87.8%<br>287,805,528:71.8%	456,815,714<br>153,715,830:69.9%<br>185,836,030:46.3%	176,108,777<br>87,303,321:39.7%<br>99,574,281:24.8%	928,402
8	79,839,368	79,235,340	99.2	72,101,480 (90.3%)	66,111,100	82.8	7.5	3,785,292,467<br>192,648,745:87.6%<br>286,001,958:71.3%	462,736,588<br>152,132,738:69.2%<br>184,144,370:45.9%	178,899,044<br>85,336,402:38.8%<br>97,764,672:24.4%	901,282
11	80,606,850	79,983,248	99.2	72,984,476 (90.5%)	66,390,479	82.4	8.2	3,816,689,993<br>193,274,513:87.9%<br>288,264,048:71.9%	469,661,072<br>154,846,432:70.4%<br>187,620,091:46.8%	184,012,071<br>89,175,433:40.5%<br>101,972,930:25.4%	899,651
15	78,831,742	78,270,016	99.3	71,376,195 (90.5%)	65,748,640	83.4	7.1	3,642,592,594<br>191,939,967:87.3%<br>288,353,099:	492,920,017<br>148,673,037:67.6%<br>182,904,068:45.6%	182,685,580<br>77,773,522:35.4%<br>90,725,448:22.6%	973,626
17	78,623,654	77,917,184	99.1	70,824,111 (90.1%)	65,850,568	83.8	6.3	3,654,313,645<br>191,881,274:87.2%<br>289,032,173:72.1%	491,659,101<br>148,449,478:67.5%<br>182,351,364:45.5%	179,855,146<br>77,754,152:35.3%<br>90,296,719:22.5%	985,122
19	74,955,626	73,955,888	98.7	67,561,119 (90.1%)	62,804,825	83.8	6.3	3,492,060,223<br>191,308,661:87.0%<br>287,877,465:71.8%	478,817,232<br>148,465,480:67.5%<br>182,090,890:45.4%	172,745,923<br>77,541,384:35.2%<br>89,730,590:22.4%	1,002,952
23	76,011,540	75,308,918	99.1	68,643,409 (90.3%)	63,310,925	83.3	7.0	3,579,437,043<br>191,078,574:86.9%<br>287,155,532:71.6%	476,431,648<br>145,815,951:66.3%<br>178,742,044:44.6%	169,131,493<br>74,032,354:33.7%<br>85,871,289:21.4%	1,133,775
24	73,091,474	72,773,373	99.6	66,551,798 (91.1%)	61,841,237	84.6	6.4	3,539,442,754<br>189,932,193:86.3%<br>281,634,287:70.2%	456,335,577<br>137,820,917:62.7%<br>168,732,711:42.1%	157,057,717<br>64,229,975:29.2%<br>75,151,566:18.7%	924,309
