# Supplementary material for: Integrating genomic resources to present full gene and putative promoter capture probe sets for bread wheat
Source: Gigascience. 2019 Jan 31;8(4):giz018. doi: 10.1093/gigascience/giz018 (PMC6461119; doi:10.1093/gigascience/giz018)
Supplement: Response_to_Reviewer_Comments_Original_Submission.pdf [file giz018_response_to_reviewer_comments_original_submission.pdf]

Author's Response To Reviewer Comments

Close

See attached document 'Response to editor and reviewers' with colour coded questions and responses. We have also attached the previous version of the manuscript that shows the corresponding tracked changes that we have made.

Close
