# Supplementary material for: Integrating genomic resources to present full gene and putative promoter capture probe sets for bread wheat
Source: Gigascience. 2019 Jan 31;8(4):giz018. doi: 10.1093/gigascience/giz018 (PMC6461119; doi:10.1093/gigascience/giz018)
Supplement: Response_to_Reviewer_Comments_Revision_1.pdf [file giz018_response_to_reviewer_comments_revision_1.pdf]

Author's Response To Reviewer Comments

Close

See attached Personal Cover letter for response to reviewers and letter to editor

Close
