# Supplementary material for: Integrating genomic resources to present full gene and putative promoter capture probe sets for bread wheat
Source: Gigascience. 2019 Jan 31;8(4):giz018. doi: 10.1093/gigascience/giz018 (PMC6461119; doi:10.1093/gigascience/giz018)
Supplement: Reviewer_1_Report_Original_Submission -- Dongying Gao — 10/24/2018 Reviewed [file giz018_reviewer_1_report_original_submission.pdf]

**Reviewer Report**

**Title: Integrating genomic resources to present full gene and putative promoter capture probe sets for bread wheat**

**Version: Original Submission** **Date:** 10/24/2018

**Reviewer name: Dongying Gao**

**Reviewer Comments to Author:**

Due to its huge genome size, high repeat content and allohexaploid genome structure, it is expensive to re-sequence the whole genome of bread wheat, therefore, development of an affordable genotyping method is valuable for wheat molecular improvement and related studies. In this manuscript, the authors used sequence capture and developed two wheat NimbleGen SeqCap EZ probe sets. As the sequencing fragments targeted mostly annotated genes and putative promoter regions, higher coverage for specific genomic regions and accurate mapping can be reached with lower price. Overall, this MS provides some interesting data and their probe sets can serve important resource for wheat genotyping. However, I feel that this MS still need to be modified and some missing information should be included, please see my comments below:
1) As the authors mentioned, the wheat genome is highly repetitive and harbors various repeats including transposons. During the development of gene probe sets, they extracted annotated gene sequences and conducted BLASTN searches against themselves as well as the chloroplast and mitochondria sequences to remove the redundant sequences. My question is that why the authors didn't search against the whole wheat genome sequences? I guess some genic sequences may be unique with their searches, but highly identical hits may be found in other regions. I would ask the authors to conduct BLASTN searches against the whole genome with their probe sequences to identify the gene sequences that shows significant sequence similarity to other sequences located in intergenic or heterochromatic regions.
2) The Figure 2 shows an example for a specific gene, but I would like to know the average coverage for all other genes at genome level. How much percent of genes cannot be sequenced and mapped to the reference genome? What areas of the unsequened genes are located at?
3) They need to compare their methods previous genotyping platform including SNP Chip and GBS, is their method cheaper, more easily operated or can reveal more variations than other methods?
4) The authors used eight CIMMYT lines for mulitplexing and sequencing analysis, but I missed the information about the eight lines, why they used these eight genotypes? What are the genomic or phenotypic variation for these lines?
5) It is not clear for me about the high confidence gene and low confidence gene, can the authors described more about these?
6) The authors defined 2-Kb region upstream of annotated genes as promoters, it may be true for many genes. However, some promoters are likely located far away from the genes that they regulate. Even promoters are located at the 2-Kb regions, how can we know what are promoter sequences? I would use "putative promoter regions" instead of "promoters".

7) The authors need to polish the MS as I can see many typos, such as "100bp" should be "100 bp" and "17Gb" should be "`17 Gb".

**Methods**

Are the methods appropriate to the aims of the study, are they well described, and are necessary controls included? Choose an item.

**Conclusions**

Are the conclusions adequately supported by the data shown? Choose an item.

**Reporting Standards**

Does the manuscript adhere to the journal's guidelines on minimum standards of reporting? Choose an item.

Choose an item.

**Statistics**

Are you able to assess all statistics in the manuscript, including the appropriateness of statistical tests used? Choose an item.

**Quality of Written English**

Please indicate the quality of language in the manuscript: Choose an item.

**Declaration of Competing Interests**

Please complete a declaration of competing interests, considering the following questions:

- Have you in the past five years received reimbursements, fees, funding, or salary from an organisation that may in any way gain or lose financially from the publication of this manuscript, either now or in the future?
- Do you hold any stocks or shares in an organisation that may in any way gain or lose financially from the publication of this manuscript, either now or in the future?
- Do you hold or are you currently applying for any patents relating to the content of the manuscript?
- Have you received reimbursements, fees, funding, or salary from an organization that holds or has applied for patents relating to the content of the manuscript?
- Do you have any other financial competing interests?
- Do you have any non-financial competing interests in relation to this paper?

If you can answer no to all of the above, write 'I declare that I have no competing interests' below. If your reply is yes to any, please give details below.

I declare that I have no competing interests.

I agree to the open peer review policy of the journal. I understand that my name will be included on my report to the authors and, if the manuscript is accepted for publication, my named report including any attachments I upload will be posted on the website along with the authors' responses. I agree for my report to be made available under an Open Access Creative Commons CC-BY license (http://creativecommons.org/licenses/by/4.0/). I understand that any comments which I do not wish to be included in my named report can be included as confidential comments to the editors, which will not be published.

I agree to the open peer review policy of the journal

To further support our reviewers, we have joined with Publons, where you can gain additional credit to further highlight your hard work (see: https://publons.com/journal/530/gigascience). On publication of this paper, your review will be automatically added to Publons, you can then choose whether or not to claim your Publons credit. I understand this statement.

Yes Choose an item.
