# Supplementary material for: Integrating genomic resources to present full gene and putative promoter capture probe sets for bread wheat
Source: Gigascience. 2019 Jan 31;8(4):giz018. doi: 10.1093/gigascience/giz018 (PMC6461119; doi:10.1093/gigascience/giz018)
Supplement: Reviewer_1_Report_Revision_1 -- Dongying Gao — 12/6/2018 Reviewed [file giz018_reviewer_1_report_revision_1.pdf]

Reviewer Report

Title: Integrating genomic resources to present full gene and putative promoter capture probe sets for bread wheat

Version: Revision 1 **Date:** 12/6/2018

Reviewer name: Dongying Gao

Reviewer Comments to Author:

1). The authors need to provide more information about the eight the eight CIMMYT wheat varieties in main text including genotypic or phenotypic divergence.
2). " pre-miRNA sequences that were annotated using IWGSC Refseq1.v1", I don't think this is a accurate description. My understand is that IWGSC Refseq1.v1 represents the genome sequence but not the program that can annotate pre-miRNA. The authors need to provide some details about how they annotate pre-miRNAs, how much longer the miRNAs are and if they are unique for specific regions.
3). Despite the authors modified their words with "putative promoter" and replace "promoter" in some places, but I can see "promoter" through entire MS. The authors need to know it is still a challenging job to annotate promoters. I would suggest that the authors can take their promoter data set to search against the the PlantProm DB (http://www.softberry.com) to give some idea about their prediction.

Methods

Are the methods appropriate to the aims of the study, are they well described, and are necessary controls included? Choose an item.

Conclusions

Are the conclusions adequately supported by the data shown? Choose an item.

Reporting Standards

Does the manuscript adhere to the journal's guidelines on minimum standards of reporting? Choose an item.

Choose an item.

Statistics

Are you able to assess all statistics in the manuscript, including the appropriateness of statistical tests used? Choose an item.

Quality of Written English

Please indicate the quality of language in the manuscript: Choose an item.

Declaration of Competing Interests

Please complete a declaration of competing interests, considering the following questions:

- Have you in the past five years received reimbursements, fees, funding, or salary from an organisation that may in any way gain or lose financially from the publication of this manuscript, either now or in the future?
- Do you hold any stocks or shares in an organisation that may in any way gain or lose financially from the publication of this manuscript, either now or in the future?
- Do you hold or are you currently applying for any patents relating to the content of the manuscript?
- Have you received reimbursements, fees, funding, or salary from an organization that holds or has applied for patents relating to the content of the manuscript?
- Do you have any other financial competing interests?
- Do you have any non-financial competing interests in relation to this paper?

If you can answer no to all of the above, write 'I declare that I have no competing interests' below. If your reply is yes to any, please give details below.

I declare that I have no competing interests.

I agree to the open peer review policy of the journal. I understand that my name will be included on my report to the authors and, if the manuscript is accepted for publication, my named report including any attachments I upload will be posted on the website along with the authors' responses. I agree for my report to be made available under an Open Access Creative Commons CC-BY license (http://creativecommons.org/licenses/by/4.0/). I understand that any comments which I do not wish to be included in my named report can be included as confidential comments to the editors, which will not be published.

I agree to the open peer review policy of the journal

To further support our reviewers, we have joined with Publons, where you can gain additional credit to further highlight your hard work (see: https://publons.com/journal/530/gigascience). On publication of this paper, your review will be automatically added to Publons, you can then choose whether or not to claim your Publons credit. I understand this statement.

Yes Choose an item.
