# Supplementary material for: Integrating genomic resources to present full gene and putative promoter capture probe sets for bread wheat
Source: Gigascience. 2019 Jan 31;8(4):giz018. doi: 10.1093/gigascience/giz018 (PMC6461119; doi:10.1093/gigascience/giz018)
Supplement: Reviewer_2_Report_Original_Submission -- Robert van Buren — 10/26/2018 Reviewed [file giz018_reviewer_2_report_original_submission.pdf]

Reviewer Report

Title: Integrating genomic resources to present full gene and putative promoter capture probe sets for bread wheat

Version: Original Submission **Date:** 10/26/2018

Reviewer name: Robert van Buren

Reviewer Comments to Author:

Gardiner et al. present two sets of high-quality targeted capture probes for genic and promoter regions of the wheat genome. Whole genome resequencing in wheat is prohibitively expensive given the large genome size (~17 Gb). GBS and other reduced representation based approaches are ill suited for wheat because of the high repeat content. The two probe sets reported here span most of the gene and promoter sequences respectively and their design using multiple reference genomes ensures the probe sets are comprehensive. These probe sets will be more expensive to use than other genotyping platforms in wheat, but the higher resolution will be useful to the community. I have a few minor suggestions that I feel will improve this manuscript prior to publication.
1. This probe set is clearly superior in terms of gene and promoter space compared to previous exon and cDNA based captures, but it is unclear how this translates to informative positions compared to previous efforts. Roughly 1.8 million high-confidence positons were comparable across the 8 sequenced CIMMYT lines with ~100 million reads per line. More discussion of how this compares to other reduced representation approaches would be useful.
2. The uniform distribution and comprehensive coverage of probes should allow for high-confidence CNV detection (provided sequencing coverage is sufficient). Were CNVs detectable in the high-coverage Chinese Spring data or the 8 sequenced CIMMYT lines?
Minor:
Page 5 line 16. The IWGSC) RefSeq.v1 sequence was recently published and should be cited here.
Version of bioinformatics tools used for read processing and alignment should be provided.
In Table 3, it would be useful to report the expected coverage with fewer reads (such as 50 million or 25 million for gene and promoter sets respectively).
The font on figure 3 is too small to read. This figure could also be reorganized so it is easier to interpret (e.g. use different colors for gene vs promoter and label the Y axis).

Methods

Are the methods appropriate to the aims of the study, are they well described, and are necessary controls included? Choose an item.

Conclusions

Are the conclusions adequately supported by the data shown? Choose an item.

Reporting Standards

Does the manuscript adhere to the journal's guidelines on minimum standards of reporting? Choose an item.

Choose an item.

Statistics

Are you able to assess all statistics in the manuscript, including the appropriateness of statistical tests used? Choose an item.

Quality of Written English

Please indicate the quality of language in the manuscript: Choose an item.

Declaration of Competing Interests

Please complete a declaration of competing interests, considering the following questions:

- Have you in the past five years received reimbursements, fees, funding, or salary from an organisation that may in any way gain or lose financially from the publication of this manuscript, either now or in the future?
- Do you hold any stocks or shares in an organisation that may in any way gain or lose financially from the publication of this manuscript, either now or in the future?
- Do you hold or are you currently applying for any patents relating to the content of the manuscript?
- Have you received reimbursements, fees, funding, or salary from an organization that holds or has applied for patents relating to the content of the manuscript?
- Do you have any other financial competing interests?
- Do you have any non-financial competing interests in relation to this paper?

If you can answer no to all of the above, write 'I declare that I have no competing interests' below. If your reply is yes to any, please give details below.

'I declare that I have no competing interests

I agree to the open peer review policy of the journal. I understand that my name will be included on my report to the authors and, if the manuscript is accepted for publication, my named report including any attachments I upload will be posted on the website along with the authors' responses. I agree for my report to be made available under an Open Access Creative Commons CC-BY license (http://creativecommons.org/licenses/by/4.0/). I understand that any comments which I do not wish to be included in my named report can be included as confidential comments to the editors, which will not be published.

I agree to the open peer review policy of the journal

To further support our reviewers, we have joined with Publons, where you can gain additional credit to further highlight your hard work (see: https://publons.com/journal/530/gigascience). On publication of this paper, your review will be automatically added to Publons, you can then choose whether or not to claim your Publons credit. I understand this statement.

Yes Choose an item.
